# A concise practical clinical guide to identifying spasticity in neurological shoulder dysfunction

**DOI:** 10.3389/fneur.2024.1440955

**Published:** 2025-01-23

**Authors:** Damon Hoad, Stephen Ashford, Ganesh Bavikatte, Rachel Farrell, Adrian Robertson, Jörg Wissel

**Affiliations:** ^1^Warwick Medical School, University of Warwick, Coventry, United Kingdom; ^2^Department of Palliative Care, Policy and Rehabilitation, Florence Nightingale Faculty of Nursing, Midwifery and Palliative Care, King’s College London, London, United Kingdom; ^3^London North West University Healthcare NHS Trust, Regional Hyper-Acute Rehabilitation Unit, Northwick Park Hospital, London, United Kingdom; ^4^Neuro-Rehabilitation Medicine, The Walton Centre NHS Foundation Trust, Liverpool, United Kingdom; ^5^Queen Square Multiple Sclerosis Centre, Department of Neuroinflammation, UCL Queen Square Institute of Neurology, Faculty of Brain Sciences, University College London, London, United Kingdom; ^6^Department of Rehabilitation, National Hospital for Neurology and Neurosurgery, UCLH NHS Foundation Trust, London, United Kingdom; ^7^Mid Yorkshire Hospitals Teaching Trust, Wakefield, United Kingdom; ^8^Neurology and Psychosomatic at Wittenbergplatz, Berlin, Germany; ^9^University of Potsdam, Potsdam, Brandenburg, Germany

**Keywords:** muscle spasticity, shoulder, arm, clinical decision-making, goal setting

## Abstract

**Background:**

Spasticity is a known complication to the hemiplegic shoulder following acquired brain injury. However, there is a current discrepancy between the diagnosis of arm spasticity and the subsequent assessment and treatment of spasticity in people with shoulder involvement.

**Objective:**

An expert panel aimed to establish a consensus and provide a simple structured approach to identifying and assessing people with spasticity of the hemiplegic shoulder.

**Methods:**

A consensus group of six spasticity experts were interviewed individually via 1-h video calls to ascertain how they assess people with arm spasticity for shoulder involvement. During an in-person meeting in March 2023, a consensus-building process was used to discuss four topics: a checklist/tool to identify shoulder involvement in upper limb spasticity, measurements/scales for assessing shoulder spasticity, ‘red flags’ for other conditions affecting the shoulder, and assessment limitations and practicalities.

**Results:**

Where there was full agreement on a topic, recommendations to overcome challenges for initial and ongoing assessment of shoulder spasticity and goal-setting were developed, and categorized as Posture, Purposeful activity and Pain (‘the three Ps’). Posture should be observed when sitting and walking, as appropriate for the person, and compared to published shoulder spasticity patterns. Purposeful activity should be assessed using a systematic approach. The presence and nature of shoulder pain should be ascertained. Recommendations on impairment- and function-related measures are given.

**Conclusion:**

This consensus guidance provides practical recommendations on identifying shoulder spasticity to support clinicians in their management of people with neurological shoulder dysfunction.

## 1 Introduction

The hemiplegic shoulder following acquired brain injury restricts movement and can cause pain; impacts negatively on mobility, hygiene, sleep, carer burden and social interaction; and affects quality of life (1–3). Clinicians must be able to identify and treat all contributing and interacting components of the hemiplegic shoulder, including consequences of impaired motor control, musculoskeletal changes, nociceptive and neuropathic pain drivers, and abnormal muscle tone (1–3).

Spasticity is a common symptom impacting the hemiplegic shoulder (1, 4, 5). Spasticity is a positive feature of the upper motor neuron syndrome, characterized by a velocity-dependent increase in muscle tone (6, 7). Disrupted control of the tonic stretch reflex results in abnormal muscle tone with movement (8). Spastic dystonia represents a spontaneous involuntary muscle activation of antagonistic muscles (7), is not velocity dependent and is characterized by the inability of the muscle to relax at rest (9, 10). It can result in pathological postures associated with pain, both at rest and when moving the arm. Furthermore, spastic co-contraction alters normal movement independent of any stretch (11). Spastic dystonia influences posture and can limit passive and active movements (12). Without effective intervention, pathological postures as a result of spastic dystonia and spasticity are then likely to contribute to soft-tissue changes and possible contracture development (11, 13). Potential differential diagnoses that should be considered when assessing patients for potential spasticity involving shoulder muscles are summarized in Table 1 and include adhesive capsulitis and complex regional pain syndrome.

**Table 1:** Differential diagnoses to consider when assessing a patient for potential spasticity affecting shoulder muscles.

Acromioclavicular joint osteoarthritis
Adhesive capsulitis
Biceps tendonitis
Contracture
Glenohumeral joint osteoarthritis
Neuropathic pain
Complex regional pain syndrome
Rotator cuff tendonitis/tendinopathy (especially supraspinatus, infraspinatus)
Shoulder impingement syndrome
Subacromial bursitis
Subluxation (where earlier low tone has later evolved to high tone)

Traditionally, several factors have limited clinician confidence in identifying and treating shoulder spasticity. In our opinion, assessment is often perceived as complicated; muscle targets for therapeutic botulinum toxin injection may be unfamiliar and injectors may be concerned about causing instability. A structured approach can provide confidence in incorporating multi-patterned botulinum toxin type A (BoNT-A) injections into the treatment plan for people with spastic hemiplegic shoulder. This is suggested as first-line treatment in the Royal College of Physicians and Toxnet guidelines (3, 14) and is the rationale for this work. However, it should also be noted that shoulder spasticity is almost never addressed in isolation and, as with any spasticity management, it is always about supporting wider management or rehabilitation. Since underlying musculoskeletal pathologies, present before or occurring simultaneously with the neurological impairment, could also trigger and perpetuate spasticity and spastic dystonia, these factors should additionally be assessed and addressed before treating the spasticity.

Unmet need is illustrated by real-world data on post-stroke arm spasticity from the Upper Limb International Spasticity Study-II (ULIS-II). This study found that 56% of people had shoulder spasticity on examination, contrasted with only 32% treated with BoNT-A injections to shoulder muscles (15). A survey of Canadian physicians in 2021 found that the injection technique and location of intramuscular BoNT-A for shoulder spasticity is highly variable among physicians and clinics (16). A clinical trial allowing clinicians treating post-stroke spasticity a choice of dose and target of BoNT-A across a range of upper limb muscles in successive treatment cycles reported only 11% of people had shoulder muscles injected in the first cycle of treatment (17), increasing to 41% of people in the fourth cycle of treatment (18). The increase was driven by the need for more effective treatment to achieve goals and was associated with improved outcomes. Such evidence highlights the need to develop a more structured approach in assessment and treatment of the shoulder.

Representative clinical trials have demonstrated the benefit of BoNT-A injections to range of shoulder movement (17, 18) and reduction of shoulder spasticity and associated pain (19, 20). Observational studies demonstrate the suitability of a pattern-based approach. In a study of 665 people with post-stroke arm spasticity, 90% of people presented with internal rotation and shoulder adduction, a characteristic of all five different arm position patterns classified in arm spasticity by Hefter et al. (21) and the two shoulder patterns subsequently described by Jacinto et al. (22).

Despite research and clinical evidence supporting use of BoNT-A in shoulder muscles for arm spasticity, shoulder involvement remains under-diagnosed and under-treated. Recent publications provide guidance on shoulder management with BoNT-A, including spasticity patterns and recommendations on muscle selection and injection techniques (14, 22–24). For details of target muscles, suggested doses for the three available BoNT-A products, and injection points, the reader is referred to Appendix 2 of the Royal College of Physicians guidelines (3). Here we build on this with additional pragmatic guidance on identification of shoulder involvement in the clinic.

An expert panel was established to address the discrepancy between identification of arm spasticity and the evaluation and treatment of spasticity in shoulder muscles. The objective was to establish consensus and provide practical guidance to ensure that clinicians treating people with arm spasticity also consider and are confident identifying shoulder involvement. As such, this work is intended to be a simple and practical introductory guide for less experienced injectors to apply in clinic, and it is beyond the scope of this article to provide a detailed diagnostic and management approach. Detailed recommendations are provided concerning the initial assessment of the shoulder for spasticity involvement, while the reader is referred to useful publications that are already available concerning the treatment and management of shoulder spasticity.

## 2 Materials and methods

A consensus group was convened through invitation based on credentials of expertise evidenced as involvement in publication or national level teaching on use of botulinum toxin for the management of shoulder spasticity. This comprised six spasticity experts with a median of 20 years’ (range 14–40 years) experience in spasticity management as well as involvement in arm spasticity studies and publications. There were two neurologists (JW and RF), two physiotherapists (SA and AR) and two rehabilitation doctors (DH and GB). Five were based in the UK and one was based in Germany. Experts were interviewed individually by the sponsor (Merz Therapeutics GmbH) via 1-h video calls to understand how they assess people with arm spasticity for shoulder involvement. Each expert was provided with the same series of open-ended questions ahead of their interview, as shown in Supplementary Table 1.

Manual transcription edited all verbal responses into distinct outputs. These were collated and deductively mapped to the structure of the four main topics that were covered: (i) development of a checklist and tool for the identification of spasticity requiring treatment in hemiplegic shoulder impairment, (ii) recommended measurements and scales for assessing shoulder spasticity, (iii) ‘red flags’ for conditions other than spasticity affecting the shoulder, and (iv) assessment limitations and practicalities.

Responses were discussed with a consensus-building process during a 6-h face-to-face focus group meeting in March 2023. The expert group were reminded of background and goals followed by facilitated discussion and consensus-building on the four main topics, chaired by a member of the expert panel. Audio and video of the meeting were recorded. Consolidated outcomes following the consensus meeting were extracted from transcripts and used to form the basis of this report.

## 3 Results

Following the consensus process, 100% agreement between the expert panel on the topics discussed during the consensus-building process was achieved, with any disagreements resolved during the consensus discussions. This resulted in the development of recommendations to overcome the challenges for initial and ongoing assessment of shoulder spasticity, in addition to strategies for goal-setting.

### 3.1 Initial assessment – ‘the three Ps’

**Figure 1:**
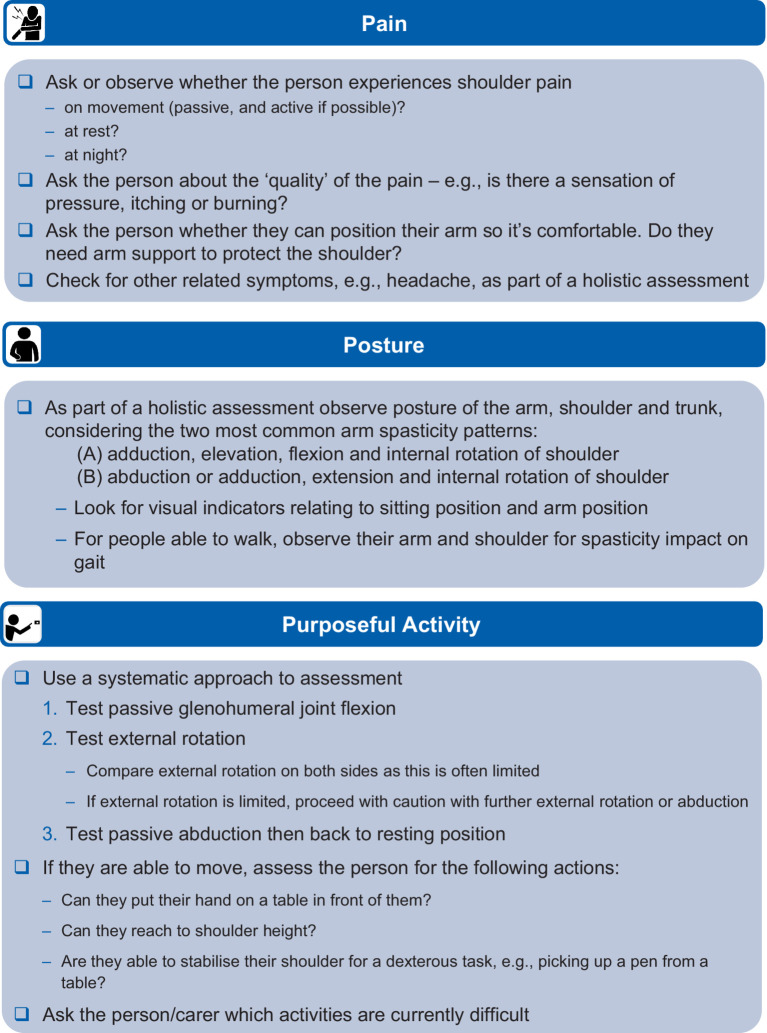
Recommendations for identifying shoulder involvement in arm spasticity.

The recommendations for initial assessment were grouped into three categories: Pain, Posture and Purposeful activity (‘the three Ps’). We recommend that the shoulder should always be included in the initial assessment of a person with arm spasticity and propose a checklist to guide the initial assessment process, as shown in Figure 1.

Pain in the shoulder should be explored with the person to understand when it is present and the nature of that pain (e.g., specific pain on movement and when in the movement it occurs). Spasticity-associated pain is usually characterized by stretch-induced or stretch-accelerated nociceptive pain (20, 25). Impingement is another reason for nociceptive pain when moving a shoulder, but impingement-related pain is usually not velocity dependent (25). It is important to always observe for pain behaviors (26, 27), and objective measures of these may be useful when assessing non-verbal patients (e.g., items of the Pain Behavioral Scale). Where a passive range of movement allows, the position of movement triggering pain may be informative or it may be possible to apply specific tests to localize musculoskeletal pain to a specific tendon (e.g., in supraspinatus tendinopathy). Osteoarthritic joint pain can be non-specific but may be worse after inactivity, with repetitive use or at the end of the day, and movements may be associated with crepitus. In a patient without voluntary active movement and pathological postures, pain approaching the end of passive range associated with harder ‘end-feel’ is likely to be related to contracture. Neuropathic pain may be associated with descriptors such as burning or needling, or there may be associated hypersensitivity or neurotrophic change.

**Figure 2:**
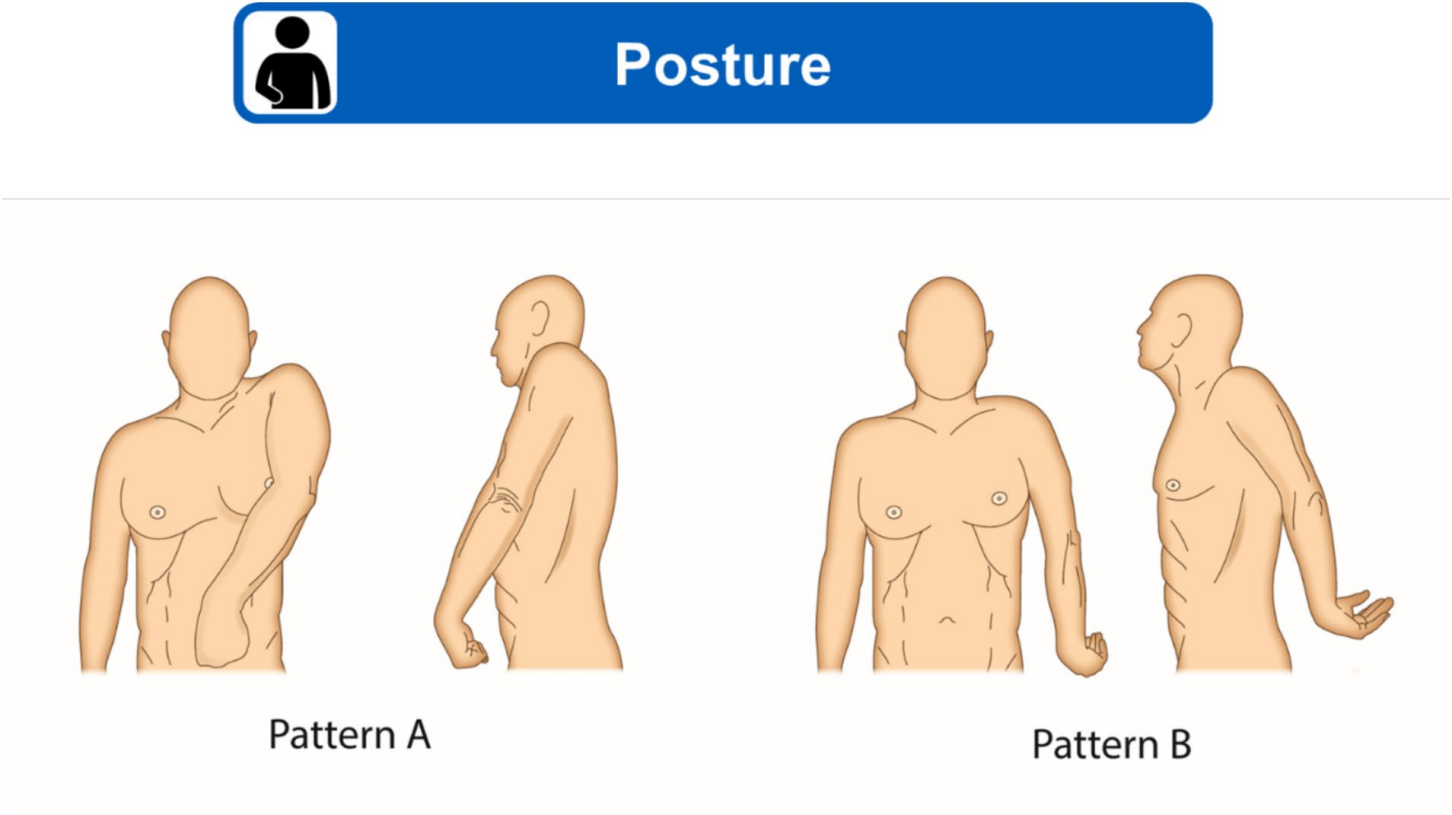
Two most common shoulder spasticity patterns. **(A)** Adduction, elevation, flexion and internal rotation of shoulder. **(B)** Abduction or adduction, extension and internal rotation of shoulder. Figure modified from Jacinto et al. (22) under a CC BY 4.0 copyright license.

Posture should be observed when sitting and, if the person is able to, when walking, or standing/assisted standing position. Posture can be compared with published patterns of shoulder spasticity; for example, the two shoulder-specific patterns reported by Jacinto et al. (22) and reproduced in Figure 2, which are the most common patterns encountered in clinical practice.

Purposeful activity should be assessed using a systematic approach, first testing passive glenohumeral flexion/extension, external rotation and passive abduction. Clinicians should feel for subluxation and, if present, should try to reduce (support) it with upward pressure before checking passive and active movement to reduce the risk of further injury during the assessment process. Care should be taken with abduction above 90 degrees to reduce risk to commonly injured rotator cuff muscles and tendons (e.g., supraspinatus and infraspinatus). If the person can move the affected limb, they should be asked to attempt functional activity that involves moving their hand into a specific position to complete a task (i.e., involving the whole arm where possible). The person and their carer should be asked about which activities they have difficulty with, including carer-assisted washing and dressing if applicable, to fully understand the impact of shoulder impairment.

In addition to ‘the three Ps’, the expert panel recommended considering alternative pathologies to enable differential diagnosis between any contribution of spasticity to shoulder impairment and the more common factors (e.g., weakness, rotator cuff damage, etc.) causing shoulder pain and dysfunction. In many cases, shoulder dysfunction and pain will be multifactorial, and the different elements (including spasticity) will need to be addressed.

### 3.2 Resulting treatment: goal-setting

A goal-setting discussion between the person with a neurological shoulder condition, their carer and the clinical team is recommended to set goals that are measurable, achievable and tailored to that person. The goals should consider specific activities that the person with a neurological condition and resulting shoulder impairment would like to be able to perform as well as the observed limitations experienced by the individual. Clinicians should be aware that nearly every upper limb goal will involve the shoulder. For example, a goal focussing on finger function is dependent on the individual being able to move their shoulder to get their hand into position.

Clinicians should be aware of the goal categories from Royal College of Physicians (RCP) guidelines in the United Kingdom and the Goal-Oriented Facilitated Approach to Spasticity Treatment (GO-FAST) tool and based on that knowledge the team should discuss individual goals with the patient and the caregiver. We recommend use of the common goal categories from the RCP guidelines (3) to aid goal-setting: (i) active function, (ii) passive function, (iii) pain relief, (iv) reduction of involuntary movements, (v) prevention of contractures/deformity, and (vi) mobility. The GO-FAST tool can be used by clinicians to provide guided assistance with goal-setting and target muscle selection for BoNT-A treatment (28). Spasticity-related goals should be set considering the three ‘P’ categories: Pain, Posture and Purposeful activity, using Goal Attainment Scaling (GAS) (29, 30) to assess the achievement of goals.

For treated individuals, we recommend looking at which goals have been set previously, and whether they were achieved or not. If they were not achieved and treatment was focused on the hand, then it is important to consider whether shoulder treatment could help to achieve their goal.

### 3.3 Relevant measures

**Figure 3:**
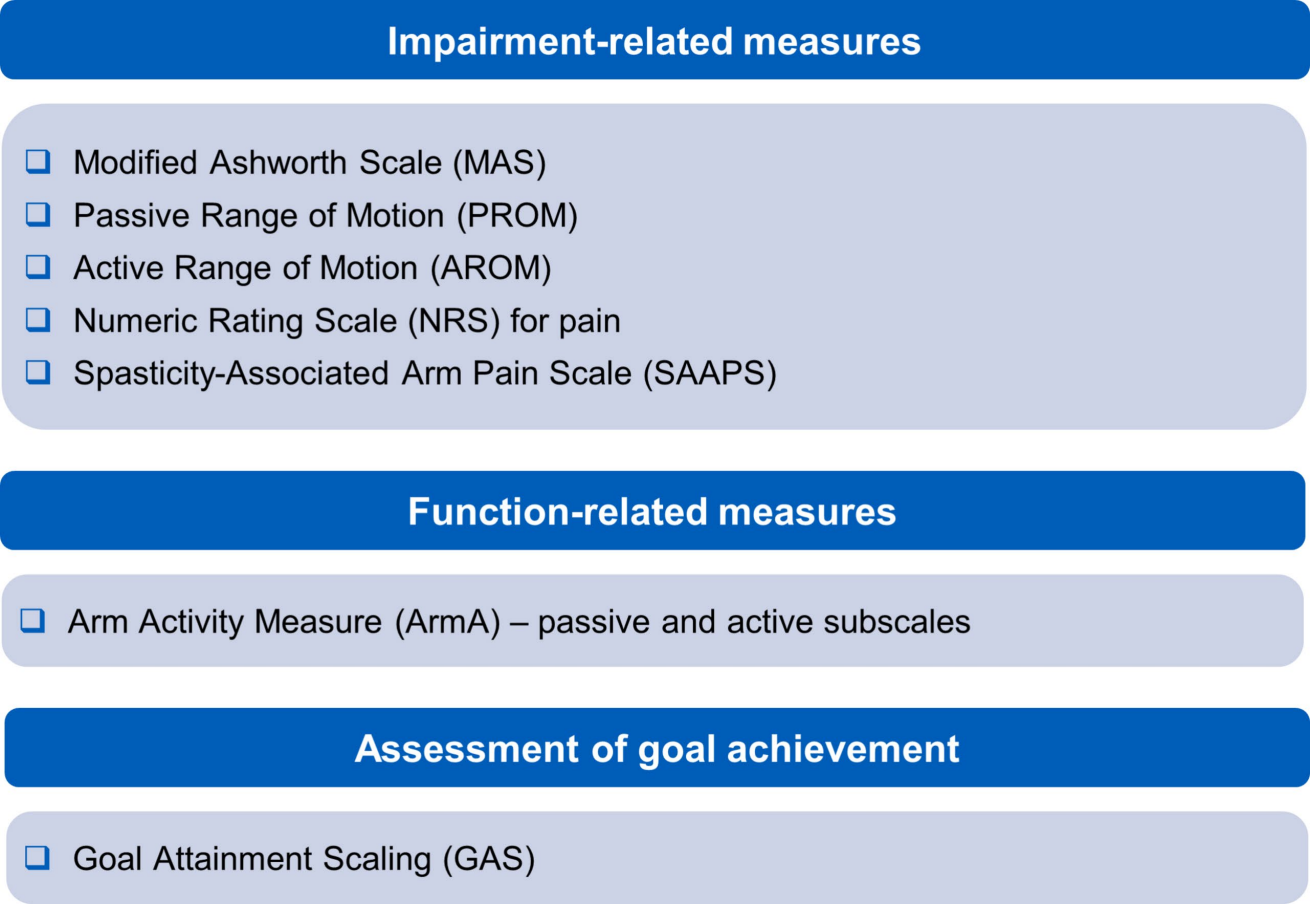
Recommended measures for managing shoulder spasticity.

In keeping with RCP guidelines (3), the authors acknowledge the usefulness of the Focal Spasticity Index measures. This comprehensive approach to measuring extent and impacts of shoulder spasticity uses a battery of relevant measures. The different elements provide measures across a range of impairment and activity frequently involved in upper limb spasticity treatment plans. Measures cover passive and active function, associated reactions, pain and quality of life. The most frequently used measures are captured in Figure 3.

Impairment-related measurements include the Modified Ashworth Scale (MAS) (31), passive and active range of motion assessments, and a numeric graphic rating scale for pain (3). Items measured may overlap with goals identified for scoring with GAS, allowing efficiency in clinic. Measuring function with the Arm Activity Measure (ArmA) (32) is particularly useful in this regard as it has the advantage of covering passive and active function, is suitable for use in spasticity and can be scored by the patient or their carers. An additional measure the authors find useful for monitoring response of pain to treatment is the Spasticity-Associated Arm Pain Scale. This records pain with shoulder abduction and external rotation, elbow, wrist and finger movements (27). It has the advantage of being quick to apply and can be used for people without verbal communication.

### 3.4 Useful management resources

The following publications provide further guidance for the clinician on managing spasticity in neurological shoulder dysfunction: (i) details on hemiplegic shoulder assessment and management by Lakra et al. (33), (ii) a framework to manage hemiplegic shoulder impairment (including pain) by Walsh et al. (34), an integrated care pathway for the management of hemiplegic shoulder pain (35), (iii) management of spasticity at the shoulder by Jacinto et al. (22) and (iv) the ViVe Algorithm, which provides a useful approach to considering diagnostic nerve blocks in the assessment and management of hemiplegic shoulder pain when spasticity first develops, by Fitterer et al. (36).

## 4 Discussion

Shoulder movement is important to nearly all upper limb treatment goals, therefore shoulder spasticity should always be considered in the management plan where shoulder impairment is identified. According to published studies (15, 17, 18) and the clinical experience of this expert panel, shoulder involvement in arm spasticity is currently underdiagnosed and undertreated. This may be due to a lack of guidance on how to identify shoulder involvement and a lack of awareness of the prevalence of shoulder spasticity following acquired brain injury. Licensed indications, muscles and total doses of BoNT-A may also play a role as well as a lack of training in localisation and guided injection of the shoulder muscles.

For the initial assessment of shoulder spasticity, ‘the three Ps’ provides a useful grouping of the consensus recommendations. Observation of posture at rest, in standing and walking will alert to commonly encountered patterns of spasticity involving shoulder muscles. Observation of pain responses during systematic examination of the shoulder will alert to spasticity-associated pain, or other contributing musculoskeletal pathologies. Passive elevation of the arm in the scapular plane should be considered, and attention should be given to external rotation and abduction, with extra care taken to avoid injuring the individual when external rotation and abduction are limited. To observe spasticity impact on purposeful activity, if the individual can move the affected limb, they should be asked to attempt actions that involve moving their hand into a specific position to complete a task. The individual and their carer should be asked about which activities they have difficulty with, including carer-assisted washing and dressing, if applicable, to help in identifying shoulder impairment.

This guidance alerts the reader to the importance of identifying shoulder spasticity when setting upper limb treatment goals and provides a simplified practical approach for first steps in embedding this into clinical practice. As the clinician’s confidence builds, they will begin to refine their examination, learning to apply specific tests for the various musculoskeletal and neuropathic processes that overlap. Detailed discussion of these is beyond the scope of this introductory approach, but frequently encountered co-existing pathologies affecting the shoulder are highlighted, and direction is given to useful resources to develop ability in diagnosis and management.

Service design is an important part of being able to deliver effective treatment plans. Limitations of staff time and clinic resources may put pressure on detailed assessment and treatment of the shoulder at the expense of the most appropriately targeted treatment. Initial telephone or virtual consultations (or pre-clinic questionnaires) can help to plan effective use of clinic time.

Neurologic shoulder dysfunction is complicated and multifactorial and consequently any successful treatment plan will likely require multiple treatment interventions (Table 2). Multi-patterned BoNT-A injections are the suggested first-line treatment for people with spastic hemiplegic shoulder (3, 14). However, treatment may be limited when the necessary equipment for guiding injections (24, 37–43) is not available.

**Table 2:** Overview of management options for spasticity affecting neurological shoulder dysfunction.

Nerve blocks
Surgery
Antineuropathic drugs
Intrarticular steroids
Physical therapy
BoNT-A

In recognition of these limitations, a simple structure has been developed by the expert panel to assist clinicians with assessment of the shoulder for spasticity involvement, and subsequent treatment, where indicated. The recommendations are designed to be applicable in a busy clinical environment, where time with the person with a neurological shoulder condition may be limited.

## 5 Conclusion

Inclusion of the shoulder in management of arm spasticity is inconsistent. A simple structure has been developed to assist clinicians with assessment of the shoulder for spasticity involvement. Shoulder movement is important to nearly all arm treatment goals; therefore, shoulder spasticity should always be considered in (or excluded from) the management plan. These consensus recommendations provide clinicians with practical guidance on identifying spasticity of the shoulder associated with stroke or other neurological conditions and support them in their approach to managing people with neurological shoulder dysfunction.

## Data Availability

The original contributions presented in the study are included in the article/Supplementary material, further inquiries can be directed to the corresponding author.

## References

[1] Wilson RD Chae J. Hemiplegic shoulder pain. Phys Med Rehabil Clin N Am. (2015) 26:641–55. doi: 10.1016/j.pmr.2015.06.007, PMID: 26522903

[2] Adey-Wakeling Z Liu E Crotty M Leyden J Kleinig T Anderson CS. Hemiplegic shoulder pain reduces quality of life after acute stroke: a prospective population-based study. Am J Phys Med Rehabil. (2016) 95:758–63. doi: 10.1097/PHM.0000000000000496, PMID: 27003204

[3] Royal College of Physicians. Spasticity in adults: management using botulinum toxin. National guidelines. (2018). Available at: https://rcp.soutron.net/Portal/Default/en-GB/DownloadImageFile.ashx?objectId=453&ownerType=0&ownerId=72228 (Accessed May 2, 2024).

[4] Ratnasabapathy Y Broad J Baskett J Pledger M Marshall J Bonita R. Shoulder pain in people with a stroke: a population-based study. Clin Rehabil. (2003) 17:304–11. doi: 10.1191/0269215503cr612oa, PMID: 12735538

[5] Lindgren I Jönsson AC Norrving B Lindgren A. Shoulder pain after stroke: a prospective population-based study. Stroke. (2007) 38:343–8. doi: 10.1161/01.STR.0000254598.16739.4e 17185637

[6] Lance JW. Symposium synopsis In: Feldman RG Young RR Koella WP, editors. Spasticity: disordered motor control. Chicago: Year Book Medical Publishers (1980). 485–94.

[7] Baude M Nielsen JB Gracies JM. The neurophysiology of deforming spastic paresis: a revised taxonomy. Ann Phys Rehabil Med. (2019) 62:426–30. doi: 10.1016/j.rehab.2018.10.004, PMID: 30500361

[8] Pandyan AD Gregoric M Barnes MP Wood D Van Wijck F Burridge J. Spasticity: clinical perceptions, neurological realities and meaningful measurement. Disabil Rehabil. (2005) 27:2–6. doi: 10.1080/09638280400014576, PMID: 15799140

[9] Denny-Brown D. The cerebral control of movement. Liverpool: University Press (1966).

[10] Lorentzen J Pradines M Gracies JM Nielsen B. On Denny-Brown’s ‘spastic dystonia’ - what is it and what causes it? Clin Neurophysiol. (2018) 129:89–94. doi: 10.1016/j.clinph.2017.10.023, PMID: 29161622

[11] Gracies JM. Pathophysiology of spastic paresis. I: paresis and soft tissue changes. Muscle Nerve. (2005) 31:535–51. doi: 10.1002/mus.20284, PMID: 15714510

[12] Burke D Wissel J Donnan GA. Pathophysiology of spasticity in stroke. Neurology. (2013) 80:S20–6. doi: 10.1212/WNL.0b013e31827624a7 23319482

[13] Trompetto C Marinelli L Mori L Pelosin E Currà A Molfetta L. Pathophysiology of spasticity: implications for neurorehabilitation. Biomed Res Int. (2014) 2014:354906. doi: 10.1155/2014/354906, PMID: 25530960 PMC4229996

[14] Reebye R Balbert A Bensmail D Walker H Wissel J Deltombe T. Module 2: nonsurgical management of spasticity. J Int Soc Phys Rehabil Med. (2022) 5:S23–37. doi: 10.4103/2349-7904.347808

[15] Turner-Stokes L Fheodoroff K Jacinto J Maisonobe P. Results from the upper limb international spasticity study-II (ULISII): a large, international, prospective cohort study investigating practice and goal attainment following treatment with botulinum toxin A in real-life clinical management. BMJ Open. (2013) 3:e002771. doi: 10.1136/bmjopen-2013-002771, PMID: 23794582 PMC3686177

[16] Kassam F Lim B Afroz S Boissonnault È Reebye R Finlayson H. Canadian physicians' use of intramuscular botulinum toxin injections for shoulder spasticity: a national cross-sectional survey. Toxins. (2023) 15:58. doi: 10.3390/toxins15010058, PMID: 36668878 PMC9866374

[17] Gracies JM Brashear A Jech R McAllister P Banach M Valkovic P. International AbobotulinumtoxinA adult upper limb spasticity study group. Safety and efficacy of abobotulinumtoxinA for hemiparesis in adults with upper limb spasticity after stroke or traumatic brain injury: a double-blind randomised controlled trial. Lancet Neurol. (2015) 14:992–1001. doi: 10.1016/S1474-4422(15)00216-1, PMID: 26318836

[18] Gracies JM O'Dell M Vecchio M Hedera P Kocer S Rudzinska-Bar M. International AbobotulinumtoxinA adult upper limb spasticity study group. Effects of repeated abobotulinumtoxinA injections in upper limb spasticity. Muscle Nerve. (2018) 57:245–54. doi: 10.1002/mus.25721, PMID: 28590525 PMC5811783

[19] Wissel J Bensmail D Ferreira JJ Molteni F Satkunam L Moraleda S. TOWER study investigators. Safety and efficacy of incobotulinumtoxinA doses up to 800 U in limb spasticity: the TOWER study. Neurology. (2017) 88:1321–8. doi: 10.1212/WNL.0000000000003789, PMID: 28283596 PMC5379931

[20] Wissel J Bensmail D Scheschonka A Flatau-Baqué B Simon O Althaus M. Post hoc analysis of the improvement in shoulder spasticity and safety observed following treatment with incobotulinumtoxinA. J Rehabil Med. (2020) 52:jrm00028. doi: 10.2340/16501977-2651, PMID: 32025741

[21] Hefter H Jost WH Reissig A Zakine B Bakheit AM Wissel J. Classification of posture in poststroke upper limb spasticity: a potential decision tool for botulinum toxin A treatment? Int J Rehabil Res. (2012) 35:227–33. doi: 10.1097/MRR.0b013e328353e3d4, PMID: 22555318

[22] Jacinto J Camões-Barbosa A Carda S Hoad D Wissel J. A practical guide to botulinum neurotoxin treatment of shoulder spasticity 1: anatomy, physiology, and goal setting. Front Neurol. (2022) 13:1004629. doi: 10.3389/fneur.2022.1004629, PMID: 36324373 PMC9618862

[23] Escaldi S Bianchi F Bavikatte G Molteni F Moraleda S Deltombe T. Module 1: pathophysiology and assessment of spasticity; goal setting. J Int Soc Phys Rehabil Med. (2022) 5:S3–S22. doi: 10.4103/2349-7904.347807

[24] Wissel J Camões-Barbosa A Carda S Hoad D Jacinto J. A practical guide to botulinum neurotoxin treatment of shoulder spasticity 2: injection techniques, outcome measurement scales, and case studies. Front Neurol. (2022) 13:1022549. doi: 10.3389/fneur.2022.1022549, PMID: 36570447 PMC9768330

[25] Yang S Chang MC. Poststroke pain. Semin Neurol. (2021) 41:067–74. doi: 10.1055/s-0040-1722641, PMID: 33469900

[26] Turner-Stokes L Disler R Shaw A Williams H. Screening for pain in patients with cognitive and communication difficulties: evaluation of the SPIN-screen. Clin Med. (2008) 8:393–8. doi: 10.7861/clinmedicine.8-4-393, PMID: 18724606 PMC4952932

[27] Fheodoroff K Kossmehl P Wissel J. Validity and reliability of the spasticity-associated arm pain scale. J Pain Manage Med. (2017) 3:127. doi: 10.35248/2684-1320.17.3.127

[28] Jacinto J Balbert A Bensmail D Carda S Draulans N Deltombe T. Selecting goals and target muscles for botulinum toxin A injection using the goal oriented facilitated approach to spasticity treatment (GO-FAST) tool. Toxins. (2023) 15:676. doi: 10.3390/toxins15120676, PMID: 38133180 PMC10748217

[29] Kiresuk T Sherman R. Goal attainment scaling: a general method of evaluating comprehensive mental health programmes. Com Mental Health J. (1968) 4:443–53. doi: 10.1007/BF01530764, PMID: 24185570

[30] Ashford S Turner-Stokes L. Goal attainment for spasticity management using botulinum toxin. Physiother Res Int. (2006) 11:24–34. doi: 10.1002/pri.36, PMID: 16594313

[31] Bohannon RW Smith MB. Interrater reliability of a modified Ashworth scale of muscle spasticity. Phys Ther. (1987) 67:206–7. doi: 10.1093/ptj/67.2.206, PMID: 3809245

[32] Ashford S Turner-Stokes L Siegert R Slade M. Initial psychometric evaluation of the arm activity measure (ArmA): a measure of activity in the hemiparetic arm. Clin Rehabil. (2013) 27:728–40. doi: 10.1177/0269215512474942, PMID: 23426566

[33] Lakra C Higgins R Beare B Farrell R Ajina S Burns S. Managing painful shoulder after neurological injury. Pract Neurol. (2023) 23:229–38. doi: 10.1136/pn-2022-003576, PMID: 36882323 PMC7616867

[34] Walsh M Ashford S Rose H Alfonso E Steed A Turner-Stokes L. Stratified management of hemiplegic shoulder pain using an integrated care pathway: an 18-year clinical cohort analysis. Disabil Rehabil. (2022) 44:5909–18. doi: 10.1080/09638288.2021.1951851, PMID: 34310224

[35] Cicely Saunders Institute of Palliative Care, Policy & Rehabilitation. Management of Hemiplegic Shoulder Pain (HSP). Available at: https://www.kcl.ac.uk/cicelysaunders/resources/toolkits/management-of-hemiplegic-shoulder-pain (Accessed March 13, 2024).

[36] Fitterer JW Picelli A Winston P. A novel approach to new-onset hemiplegic shoulder pain with decreased range of motion using targeted diagnostic nerve blocks: the ViVe algorithm. Front Neurol. (2021) 12:668370. doi: 10.3389/fneur.2021.668370, PMID: 34122312 PMC8194087

[37] Picelli A Bonetti P Fontana C Barausse M Dambruoso F Gajofatto F. Accuracy of botulinum toxin type A injection into the gastrocnemius muscle of adults with spastic equinus: manual needle placement and electrical stimulation guidance compared using ultrasonography. J Rehabil Med. (2012) 44:450–2. doi: 10.2340/16501977-0970, PMID: 22549655

[38] Picelli A Lobba D Midiri A Prandi P Melotti C Baldessarelli S. Botulinum toxin injection into the forearm muscles for wrist and fingers spastic overactivity in adults with chronic stroke: a randomized controlled trial comparing three injection techniques. Clin Rehabil. (2014) 28:232–42. doi: 10.1177/0269215513497735, PMID: 23945164

[39] Turner-Stokes L Jacinto J Fheodoroff K Brashear A Maisonobe P Lysandropoulos A. Upper limb international spasticity-III (ULIS-III) study group. Assessing the effectiveness of upper-limb spasticity management using a structured approach to goal-setting and outcome measurement: first cycle results from the ULIS-III study. J Rehabil Med. (2021) 53:10.2340/16501977-2770. doi: 10.2340/16501977-2770, PMID: 33206198 PMC8772374

[40] Fheodoroff K Ashford S Jacinto J Maisonobe P Spurden D Grandoulier AS. Turner-Stokes L, et al. Predictors of real-world response in adults treated with botulinumtoxin-A for upper limb spasticity. J Int Soc Phys Rehabil Med. (2024) 7:24. doi: 10.1097/ph9.0000000000000030, PMID: 39703727

[41] Royse CE Sha S Soeding PF Royse AG. Anatomical study of the brachial plexus using surface ultrasound. Anaesth Intensive Care. (2006) 34:203–10. doi: 10.1177/0310057X0603400212, PMID: 16617641

[42] Daniels SP Viers CD Blaichman JI Ross AB Tang JY Lee KS. US-guided musculoskeletal interventions of the Body Wall and Core with MRI and US correlation. Radiographics. (2021) 41:2011–28. doi: 10.1148/rg.2021210050, PMID: 34623945

[43] Rha DW Han SH Kim HJ Won SY Lee SC. Ultrasound-guided lateral approach for needle insertion into the subscapularis for treatment of spasticity. Arch Phys Med Rehabil. (2012) 93:1147–52. doi: 10.1016/j.apmr.2012.02.017, PMID: 22503934

