# Supplementary material for: A concise practical clinical guide to identifying spasticity in neurological shoulder dysfunction
Source: Front Neurol. 2025 Jan 23;15:1440955. doi: 10.3389/fneur.2024.1440955 (PMC11800359; doi:10.3389/fneur.2024.1440955)
Supplement: Supplementary file 1 [file Data_Sheet_1.PDF]

Supplementary Table 1. List of questions asked during individual interviews prior to the consensus meeting

Q1	*You have a patient with upper limb spasticity and it's the first time you've met them. What are the steps you go through with that patient in their initial consultation to guide your treatment decisions?*
Q2	*What types of goals would a patient or carer have that would make you think about shoulder assessment?*
Q3	*How do you identify which patterns are involved in upper limb spasticity?*
Q4	*What measures would be incorporated into assessment – e.g. scales?*
Q5	*Thinking particularly about shoulder spasticity, what questions do you ask a patient which help you determine if there is shoulder involvement?*
Q6	*What are the key things that you listen and look for to identify shoulder involvement? Thinking about verbal and physical clues.*
Q7	*What movements do you ask a patient to perform to rule out/in shoulder involvement? Active or passive movements.*
Q8	*Are there any resource limitations in your clinic which limit investigation of shoulder involvement when assessing a patient with upper limb spasticity?*
